# Cylindrical single-wall and double-wall structures with or without internal water subjected to underwater shock loading

**DOI:** 10.1016/j.heliyon.2024.e26930

**Published:** 2024-02-26

**Authors:** Vernajo P. Macapagal, Young W. Kwon, Jarema M. Didoszak

**Affiliations:** Department of Mechanical and Aerospace Engineering, Naval Postgraduate School, Monterey, CA 93943, USA

**Keywords:** Underwater shock, Carbon fiber composite, 3-D printing, Cylinder, Water-back, Air-back

## Abstract

A series of experimental studies were conducted for cylindrical structures subjected to underwater shock loading to understand their dynamic responses and failure characteristics. All tests were performed inside an anechoic water tank. The submerged test cylinders were freely suspended, and an underwater shock loading was generated by the Compressed Air Shock Pipe Underwater Release (CASPUR) system. Cylinders were made of two different materials. The first group of cylinders was fabricated from carbon fiber and resin using the filament winding technique. The winding angles were ± 45° resulting in the same properties along axial and hoop directions. The second group of cylinders was constructed using a 3-D printer with polylactic acid (PLA) material. The 3-D printed cylinders had an orthotropic material property with different values in the axial and hoop directions. Both single-wall and double-wall cylindrical structures were tested. The latter consisted of two concentric cylinders of different diameters with uniform spacing between them. In addition, within the single-wall cylinders and the annuli of double-wall cylinders, the water fill was varied at 0%, 50%, or 100%. Pressure and strain gages were used to measure the shock pressure and deformation of the cylinders. The number of cylinders such as single-wall or double-wall and the internal water resulted in significant effects on the measured dynamic response (i.e., strain gage response) as well as the failure loading and failure characteristics including major failure locations. Internal water reduced the strain on the cylinders and made them withstand greater shock loading for both single-wall and double-wall cylinders.

## 1 Introduction

The usage of polymer composite materials is gaining way throughout various engineering fields including naval engineering. Polymer composite materials have a high strength-to-weight ratio and are effective against corrosion which is one of the key issues in marine structures. As a result, extensive research has been conducted on composite materials and structures to understand their durability and resistance to various forms of loading.

There are two major types of dynamic loading: impact and shock loading. One of the most studied topics is a low-velocity impact because such loading can damage composite materials and structures [[1], [2], [3], [4], [5], [6], [7]]. The analysis of impact loading of structures within the water needs to consider fluid-structure interaction (FSI) because the density of water is comparable to that of polymer composite materials [[8], [9], [10], [11], [12], [13], [14], [15], [16], [17]].

The effect of FSI is very different between impact and shock loading because the former is applied at local areas of structures while the latter encompasses the whole structure. Underwater explosion (UNDEX) is a typical cause of shock loading within the water and needs to consider FSI when the loading is applied to an underwater structure. Because military vessels could be subjected to UNDEX, research was performed on composite structures subjected to UNDEX [[18], [19], [20], [21], [22], [23], [24], [25], [26], [27]]. Both air-back and water-back plate structures were examined through mostly experimental means. Recently, both experimental and numerical studies were conducted for both air-back and water-back conditions to compare and better understand their results [27].

Almost every study concerning impact and shock loading considered either composite or sandwich flat plates. There has been much less research on shell structures such as cylindrical or spherical shapes [24,28]. Furthermore, a limited study has been conducted on the dynamic response of multiple structures coupled with fluid between them [[28], [29], [30]]. Those prior studies used impact loading.

The objective of this research was to investigate dynamic responses and failure of various cylindrical structures subjected to underwater shock loading including fluid coupling. Test cylinders were made of filament-wound carbon fiber composite (CFC) as well as 3-D printed polylactic acid (PLA). The polymer materials are light and corrosion-resistant, so they are considered a good choice to replace conventional metallic materials for underwater structures. Both CFC and PLA materials have greater strength in the fiber or the printing direction resulting in anisotropic material behavior. In addition, both materials are either brittle or quasi-brittle, so they behave very differently from conventional isotropic metallic materials.

Both single-wall and double-wall cylinders were considered. Additionally, the single-wall cylinders and the annuli of the double-wall cylinders were filled with various amounts of water, 0%, 50%, or 100%, which related to empty, half-full, or completely full conditions to understand the effect of the internal water on dynamic responses and failure of those cylinders. The next section describes the experimental setup, including the fabrication of test specimens and placement of strain gages. The subsequent section presents an overview of the test facility and a description of how to generate the underwater shock pressure, data acquisition, and testing procedure. Finally, test results are presented and discussed in the following section, and the summary and conclusions are provided.

## 2 Experimental set-up

The experimental setup is organized into three parts. The first part discusses the fabrication of each composite material; the second part discusses post-fabrication and requirements for testing; and the last part goes over the cylinder support structure redesign.

### 2.1 Fabrication

Cylinders were fabricated using CFC and PLA. These are polymeric materials selected to replace conventional metallic materials for some underwater structures such as unmanned underwater vehicles. The CFC cylinders were fabricated using the filament winding process. The process of creating a CFC composite cylinder was the wet winding process which uses dry CFC fibers and the resin bath. The fibers become wet by going through the resin bath before being wound on a mandrel. Because the resin contains a hardener, the wound CFC fibers are set on the mandrel along with time. The dry fiber used in this study is “TORAY” T700S with ProSet© M1002 epoxy resin and hardener. Material properties for the CFC are provided in Table 1.

**Table 1:** CFC cylinder material properties.

*E*_*L*_ (GPa)^a^	*E*_*T*_ (GPa)^a^	*G*_*LT*_ (GPa)	*G*_*TT*_ (GPa)	ν_LT_	ν_TT_	*ρ* (kg/m^3^)
139	7.35	2.18	0.432	0.236	0.216	1485

a *L* and *T* denote the longitudinal and transverse directions, respectively.

**Fig. 1:**
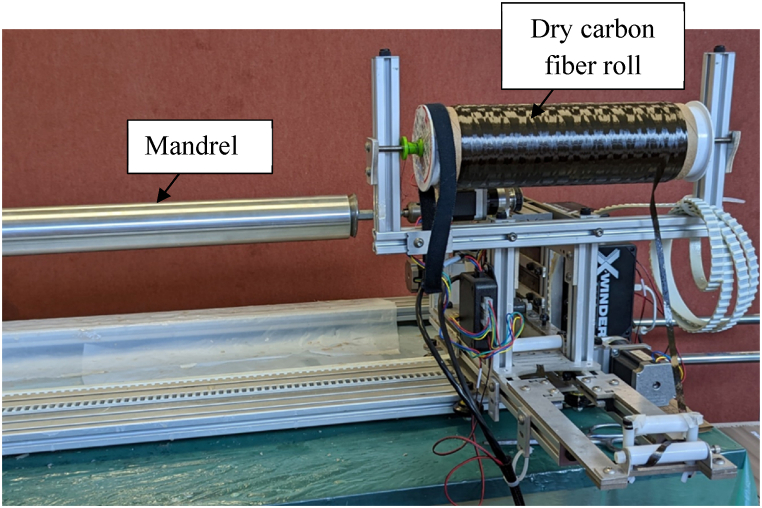
X-winder filament winding machine.

Fig. 1 displays the winding machine with a dry fiber roll loaded and a cylinder mold on the mandrel. A total of 10 composite cylinders were fabricated with six cylinders at a 15.2 cm (6 in) diameter and four cylinders at a 12.7 cm (5 in) diameter. The cylinder mold for both diameters was designed to produce a 66 cm (26 in) sample length.

The amount of resin mixture per cylinder varied with the number of layers as well as the room temperature at the time of winding. Table 2 provides the total amount of resin to hardener mixture in weight used based on the number of layers for each cylinder diameter.

**Table 2:** Resin mixture based on diameter and number of layers.

Cylinder Diameter	Number of Layers	Resin-to-Hardener Weight Ratio^a^
15.2 cm (6 in)	One	200:48
15.2 cm (6 in)	Two	600:144
12.7 cm (5 in)	Two	400:96
12.7 cm (5 in)	Three	500:120
12.7 cm (5 in)	Four	1000:240

a Measured in grams.

**Fig. 2:**
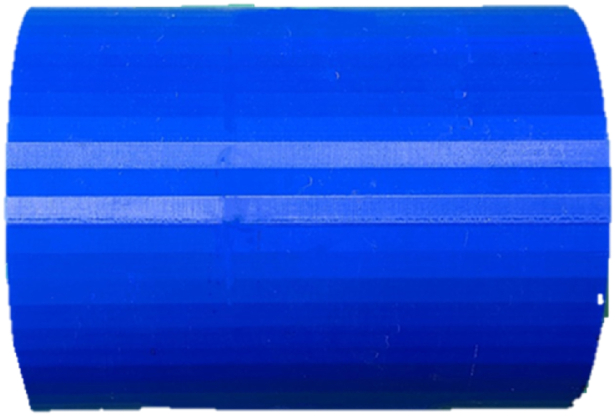
PLA cylinder sample, 15.2 cm (6 in) diameter.

The PLA cylinder samples were designed using the SolidWorks computer application, then saved in the stereolithography file format to be able to be read by the slicer program for printing. The slicer program used was Ultimaker Cura® 4.7 Slicer which coordinates with the fully automated 3-D printer Ultimaker S5 Pro. All PLA samples were printed using the print settings given in Table 3 for controlled production. The print duration of each cylinder spanned between 21 and 24 h for a finished product and ranged from 120 g to 145 g of the filament material used. Fig. 2 displays a finished 15.2 cm (6 in) diameter PLA cylinder. Table 4 shows the inner diameter and thickness measurements for both composite materials.

**Table 3:** PLA sample print settings for ultimaker Cura®.

Print Setting	Value
Layer Height (mm)	0.2
Line Width (mm)	0.35
Wall Thickness (mm)	1
Wall Line Count	1
Infill Density (%)	100
Infill Pattern	Lines
Print Temperature (°C)	185
Build Plate Temperature (°C)	55
Print Speed (mm/s)	45
Build Plate Adhesion Type	Brim

**Table 4:** CFC and PLA composite cylinder dimensions.

Sample	Inner Diameter	Thickness
CFC(1)	152 mm (6 in)	1.78 mm (0.07 in)
CFC(2)	152 mm (6 in)	6.99 mm (0.275 in)
CFC(3)	152 mm (6 in)	6.99 mm (0.275 in)
CFC(4)	89 mm (3.5 in)	1 mm (0.04 in)
PLA(1)	152 mm (6 in)	2.4 mm (0.09 in)
PLA(2)	152 mm (6 in)	2.4 mm (0.09 in)
PLA(3)	152 mm (6 in)	2.4 mm (0.09 in)
PLA(4)	127 mm (5 in)	2.4 mm (0.09 in)

### 2.2 Post-fabrication and requirements

The location of the strain gages around the outer and inner cylinders was selected to capture the FSI of the shock loading throughout the body of the cylinders. Test sample structures were limited to a maximum of four strain gage rosettes in total because of the limitations of the data acquisition system used, which is described in the next section.

For single-walled cylinders, the strain gage rosettes were attached to the outer surface at locations dividing it into four quadrants along the circumference. All of them were at the mid-length of the cylinders. The front of the cylinder is the high-impact zone where it is directly in the line of the shock loading. This is the location of the first strain gauge rosette. In the right axial view, following the circumferential direction clockwise, the remaining three strain gage rosettes are in ascending order: top, posterior, and bottom. These strain gage rosettes are placed on the outer surface for ease of access as well as application.

**Fig. 3:**
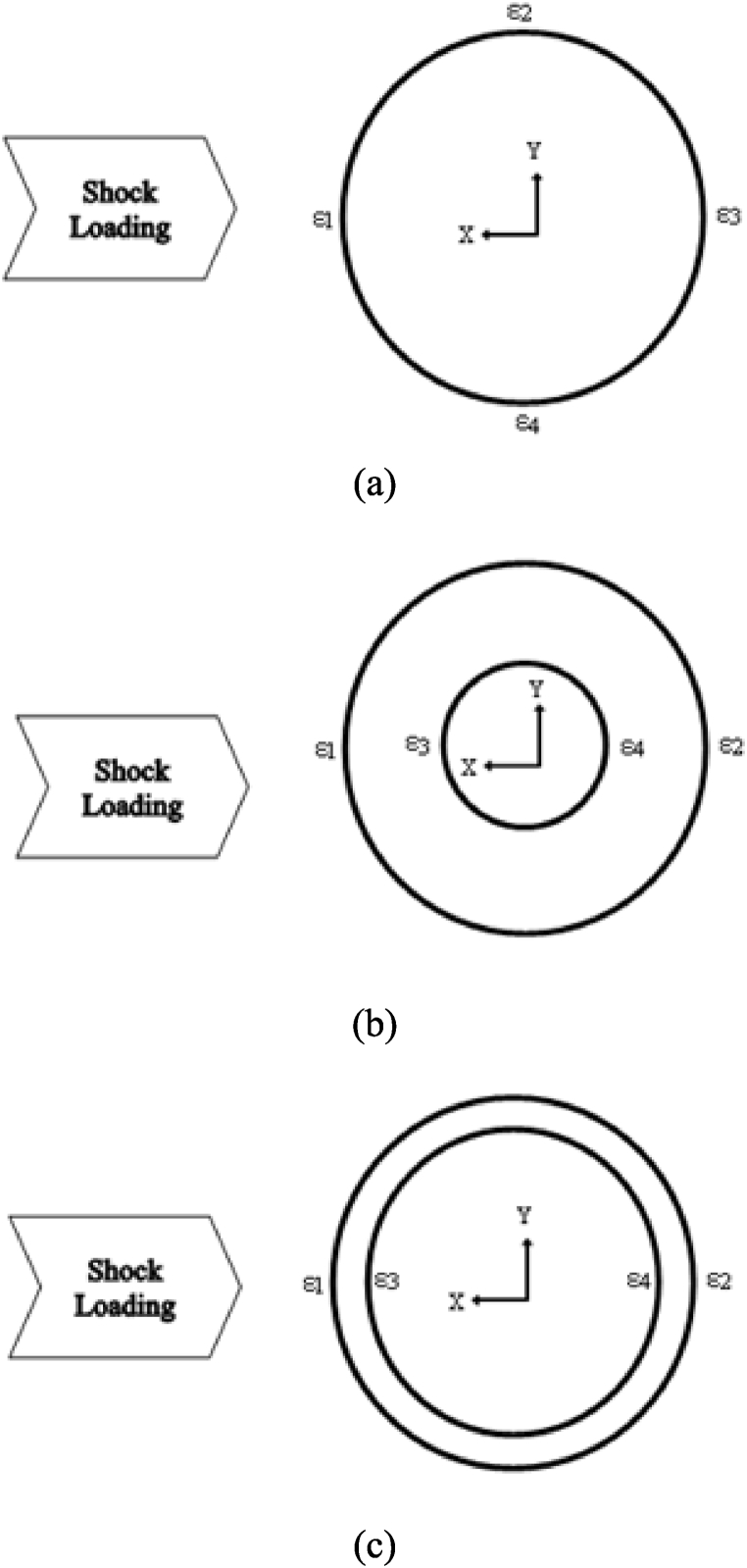
Strain Gauge placement for (a) single-walled cylinders (b) double-walled CFC cylinders (c) double-walled PLA cylinders.

Double-walled cylinders include more coordination between the inner and outer cylinders. The outer wall for both CFC and PLA materials has two strain gage rosettes attached on the outer surface; the first is placed on the front towards the shock loading, and the second at the opposite side of the load. In addition, two strain gage rosettes were also placed on the inside surface of the inner-wall cylinders in the same locations as the outer cylinders. Fig. 3(a–c) shows the strain gage rosette locations of single-wall and double-wall for CFC and PLA cylinders.

The attachment process for strain gage rosettes consisted of sample surface preparation, epoxy application, soldering wire connections and finally water-proofing the strain gage rosettes. Due to the submerged environment, water-proofing the strain gage rosettes is a necessary step for data acquisition. A silicone-based room temperature vulcanizing (RTV) coating was used on top of the strain gage rosette and wire connections to ensure the strain gages had no contact with water and were pliable enough for the natural movement of the gages.

### 2.3 Cylinder support structure redesign

**Fig. 4:**
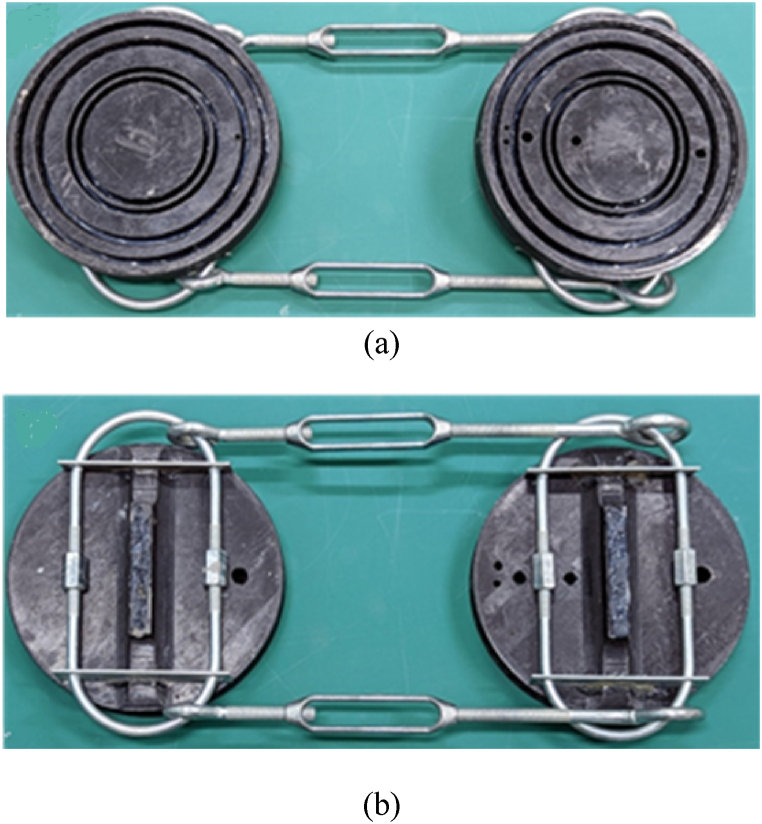
(a) Inside and (b) outside of the end caps for cylinder.

The designed test rig holding the test sample required the same submerged depth as the underwater shock loading system to align the loading with the test specimen. To solve this issue, the rig uses a drop line made of vinyl-covered steel wire attached to a hold bar, which is an anchor point for the suspended weight just below the test sample. The added weight is to counter the buoyancy force when the cylinders are filled with air internally as well as to maintain stillness once the test rig is lowered underwater. Turnbuckles and U-bolts are latched on to tighten the end caps together and maintain a closed boundary condition. Fig. 4 (a, b) shows the end caps. The top image in the figure shows the inner face of the end caps that are to be in contact with the cylinders, and the bottom image shows the outside with the attachment.

A watertight specimen is key to ensuring internal water levels are accurate and the surrounding water is kept out of the cylinders. Multiple trials of rubber gaskets, silicone coatings, and sealant tapes were used in the test runs, but the most water-tight seal is a strip of leather covered in rubber compound also known as tire plugs. They can fit between the grooves in the end caps and adhere to the cylinders well enough to keep water in and out of the samples.

## 3 Experimental procedures

### 3.1 Underwater shock testing equipment

The underwater shock testing was conducted inside an anechoic water tank at the lab of the Naval Postgraduate School (NPS). The tank has a cubic shape of 3.048 m (10 ft). The common test for underwater shock loading is conducted using an explosive detonated underwater. Dealing with an actual explosive is not desirable in a university laboratory setting. In addition, explosives do not produce very repeatable shock pressure. Thus, the Compressed Air Shock Pipe Underwater Release (CASPUR) system was locally designed, fabricated, and installed inside the anechoic water tank to generate repeatable shock waves. The compressed air supply system provides the necessary pressure to CASPUR. The compressed air supply system includes a Type K high-pressure steel tank having 17.93 MPa (2600 psi) of industrial air grade with an internal volume of 6.43 m^3^ (1.65 ft^3^). A stainless steel braided flexible hose connection measuring 9.14 m (30 ft) transports the compressed air from the tank to the shock assembly.

**Fig. 5:**
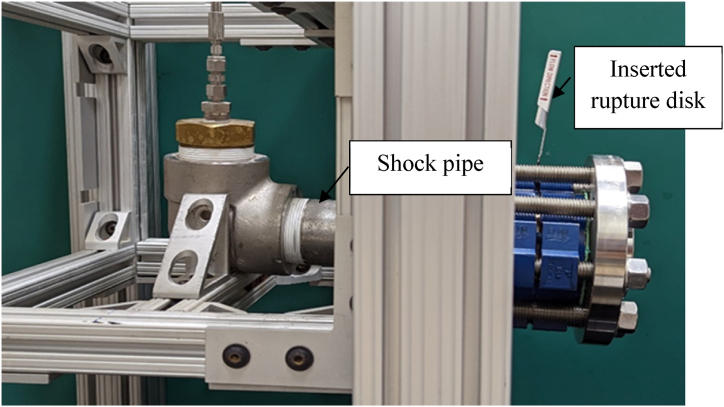
Rupture disk assembly on the shock test rig.

The shock assembly consists of a rupture disk designed to burst at 2068.4 kPa (300 psi) with a ±5% tolerance held in between a disk holder. The rupture disk manufactured by Zook® is a 5.08 cm (2 in) cross-scored forward-acting stainless steel metal rupture disk under their FAX series. The pre-torque type FAH series disk holder, also by Zook®, has an inlet and outlet flange with high-strength caps crews to fasten the rupture disk in place which is then bolted onto the shock pipe elbow. Fig. 5 shows a rupture disk assembled in the CASPUR system.

### 3.2 Data acquisition system

Experimental data were recorded and organized through the data acquisition (DAQ) system which is made up of a computer processor, data collection programs, and sensors. Raw data were managed using the National Instruments™ (NI) PXIe-1071 processor with a DAQ card, model NI PXIe-6358, which gathers the pressure and strain signals from the sensors. The NI MAX software is the computer program available in the processor to equalize the strain gage input channels through the Wheatstone Quarter Bridge I potentiometers. In addition, the NI Signal Express (2015) program, performs the processing and recording of the data. A total of 200 k sample readings was set with a sampling rate of 20 kHz to capture the full dynamic response.

### 3.3 Sensors

The pressure sensors utilized in this research were the Tourmaline ICP® underwater blast sensor, manufactured by PCB® Piezotronics, equipped with a maximum pressure rating of 344,750 kPa (50,000 psi) [22]. Strain gage rosettes, specifically K-series Tee Rosettes, manufactured by Omega® were used for the CFC cylinders. In addition, general-purpose three-element 45° rectangular rosettes by Micro Measurements® were used for the PLA cylinders. The only reason for the change in strain gages is supply availability during the tests. The wire connection from the strain gage on the sample to the DAQ measures 228.6 cm (90 in) in length for both CFC and PLA cylinders.

### 3.4 Video recording

**Fig. 6:**
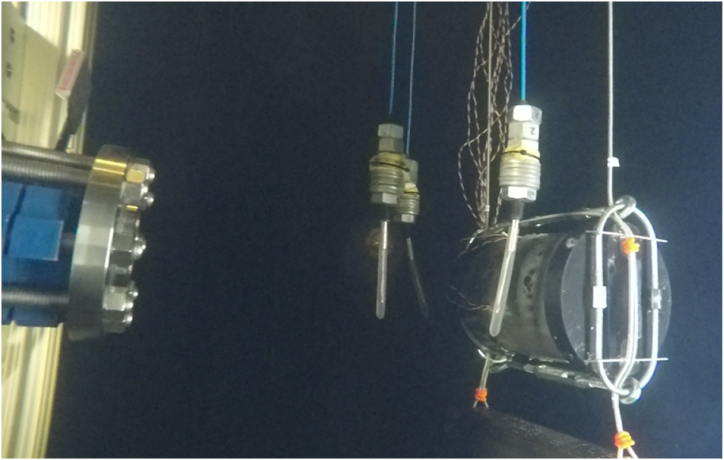
Underwater capture of experimental test setup.

A GoPro HERO® 4 camera device was used to record the underwater images during testing for FSI with the underwater shock loading. The viewing angle was from a profile view of the cylinder to capture the shock loading impact on the samples. Fig. 6 shows an underwater capture of the shock assembly aligned with the underwater pressure sensors and the cylinder sample in the test rig.

### 3.5 Testing procedures

All cylinders used the same set of end caps, so the following procedures apply for both CFC and PLA cylinders at all diameters. Regardless of the structure configuration, single-walled or double-walled, the prepared sample(s) is placed on one side of the test rig ensuring full contact between the cylinder and end cap. If applicable, the inner cylinder wire connection on the strain gage rosettes is fed through the opening of the end cap while guiding the wire away from the edges of the sample to avoid accidental cutting or crimping. Then, the other side of the end cap is secured to the opposite side of the sample.

**Fig. 7:**
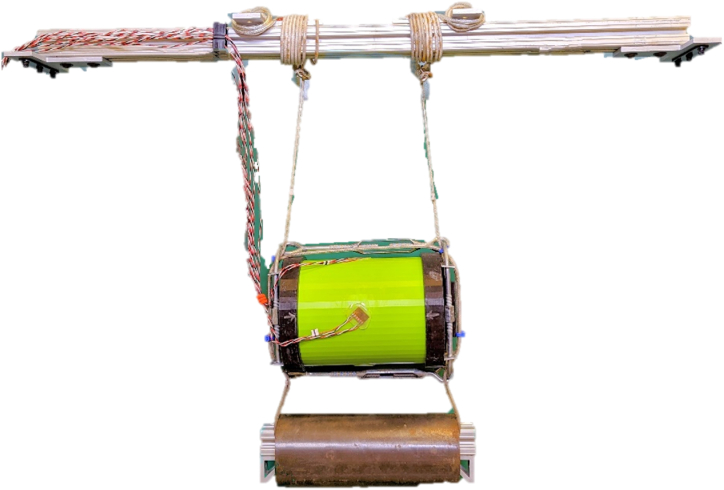
PLA cylinder on composite test rig with the hold bar.

The last step is to tighten the end caps together using the turnbuckles, at a balanced thread turn, for an even distribution throughout the sample boundaries. Turnbuckles are positioned on the top and bottom of the sample for security and to minimize shock load interference. Fig. 7 shows a ready sample on the composite test rig with the drop line retracted on the hold bar. For the test runs involving water levels within the annulus, another opening on the end cap is available to fill to the necessary water levels, and RTV sealant tape is used to plug the opening to the submerged environment.

**Fig. 8:**
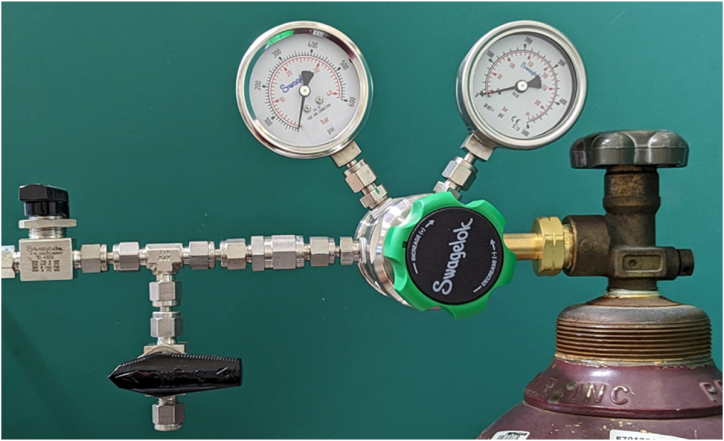
Compressed air supply system control valves.

After the rupture disk assembly, the CASPUR system is then lowered underwater to conduct a leak test before an actual test. Once the system passes the leak test, the pressure is increased gradually until the rupture of the disk using control valves as shown in Fig. 8 which displays the valves used connected to the compressed air tank that controls the air low.

The anechoic tank has six I-beams that run parallel to the directed shock wave from the loading source and supports six removable plywood pieces that serve as a tank deck cover. A test cylinder is submerged exactly 1.98 m (78 in) below the beams using a vinyl-covered steel wire anchored by a hold bar on the beams. Pressure sensors are then lowered at specified standoff distances from the center of the rupture disk.

**Fig. 9:**
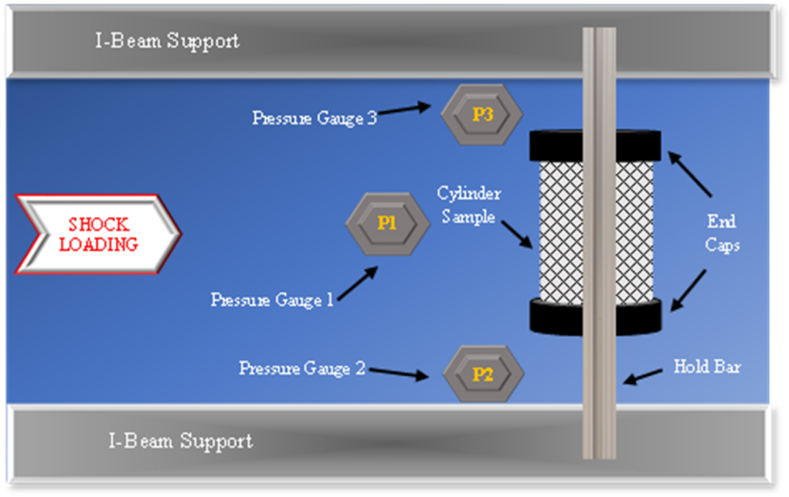
Pressure sensor diagram for shock load testing top view.

Three pressure sensors are used in a triangular formation with the first pressure sensor (P1) closest to the rupture disk, and the second pressure sensor (P2) along with the third pressure sensor (P3) positioned at the same radial distance from the shock loading as the test sample. This configuration is to take note of the maximum pressure from P1 and to consider the radial shock wave of the loading source. Fig. 9 shows the pressure sensor diagram for a shock load test.

## 4 Results and discussion

CFC composite and PLA cylinders are fabricated, submerged, and tested under directed underwater shock loading using the CASPUR system. This section will discuss the pressure loading and dynamic responses of two different cases for these cylinders. The arrangement of conducted experiments allows multiple test runs on the same cylinders. Unfortunately, this type of testing method has a potential flaw, whereas microfractures may occur during the initial shock loading tests and weaken the fibers on the surface or internally. To manage this concern, both visual and physical examinations of the outer surface and inner diameter were conducted before the next test run, and underwater video documentation was recorded to corroborate findings.

Early trial samples were used to assess the tolerance of the composites at several stand-off distances with water and/or air-filled within. For double wall cylinders, 100% water level is tested at a far stand-off distance first. If a fracture or rupture is not detected, then the water level is lowered to 50% and tested again at the same stand-off distance. If no fracture or rupture occurs after the run with the 0% water level, then the cylinders are moved closer to the shock source. With that preliminary data found, a starting stand-off distance is established for CFC and PLA cylinders at 0.45 m (1.47 ft) and 0.75 m (2.46 ft), respectively.

The naming convention for the cylindrical samples is as follows: the single number in parenthesis indicates a single-wall cylinder structure and a pair of numbers indicates a double-wall structure. Here the first number is the outer cylinder, and the latter is the inner cylinder. Each cylinder number refers to that in Table 4. The water levels specify the amount of water internal to the structure for the single-wall cylinders and within the annuli for the double-wall cylinders.

### 4.1 Pressure loading analysis

Three pressure sensors are configured in the same triangular formation throughout the experimental process as shown in Fig. 9. The largest pressure reading for each test run is P1, compared to P2 and P3, primarily because the stand-off distance for P1 is half of those of P2 and P3 unless mentioned otherwise. Additionally, P2 and P3 pressures are averaged given that these sensors are positioned at the same radial distance from the shock source portrayed.

**Fig. 10:**
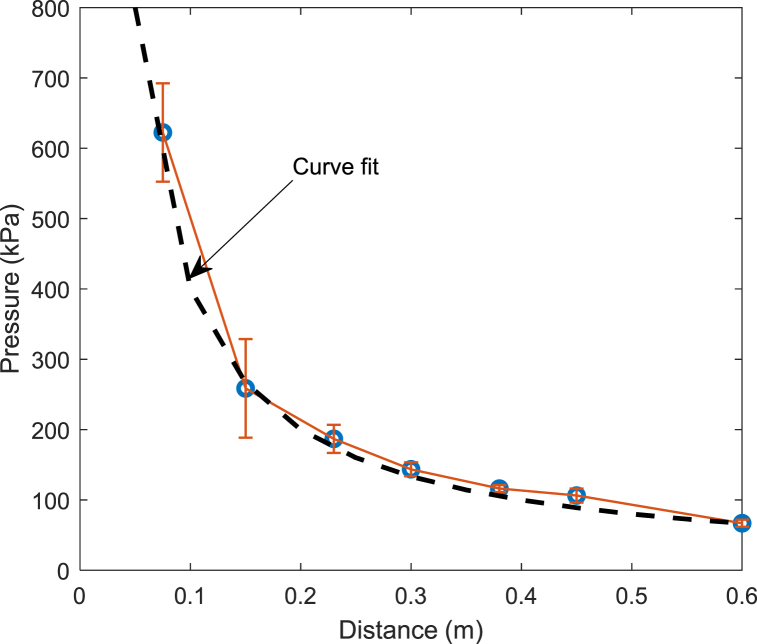
Peak pressure vs. stand-off distance plot.

Measured maximum pressure as a function of the stand-off distances with mean and standard deviation is presented in Fig. 10. As predicted, the maximum shock loading decreased with the stand-off distance in a nonlinear manner [17,24]. The peak pressure was curve-fitted accurately using the following equation:

(1) $$P=\frac{40}{6}$$

where *p* is the maximum pressure in kPa, and *R* is the stand-off distance to the pressure gage in meters. The equation suggests that the maximum pressure was inversely proportional to the stand-off distance.

**Fig. 11:**
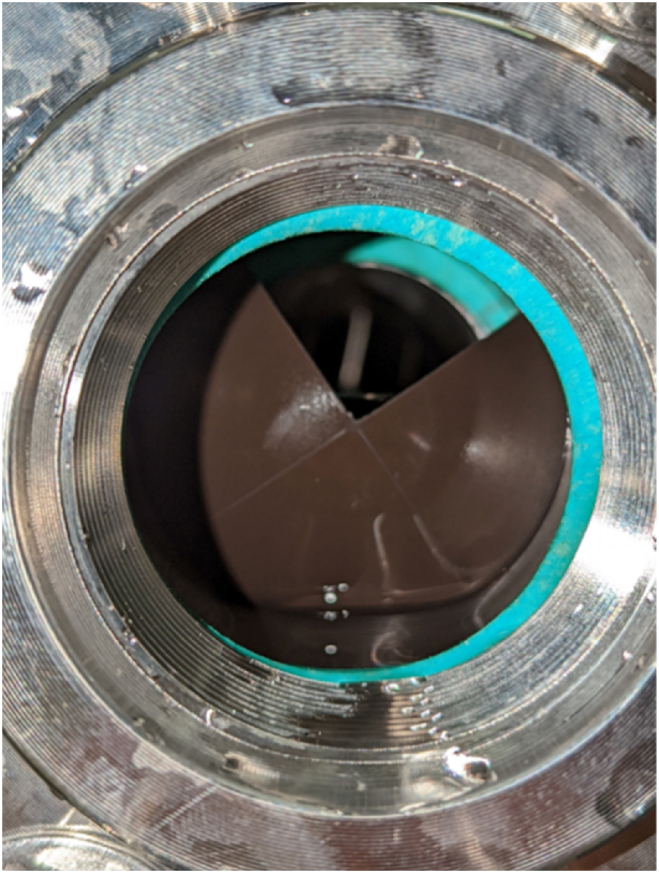
Zook® rupture disk post-test run with one petal opened.

One of the reasons for the scattered maximum pressures was the difference in the number of petals, which is noted as each of the quadrants on the rupture disk. During the experiments, the rupture disks showed only one or two petals at a time, and rarely all four which would mean that the disk was fully ruptured. However, all the rupture disks failed consistently at a pressure close to the designated value of 2.06 MPa (300 psi). Fig. 11 displays an example of a rupture disk post-test run with one petal opened.

**Fig. 12:**
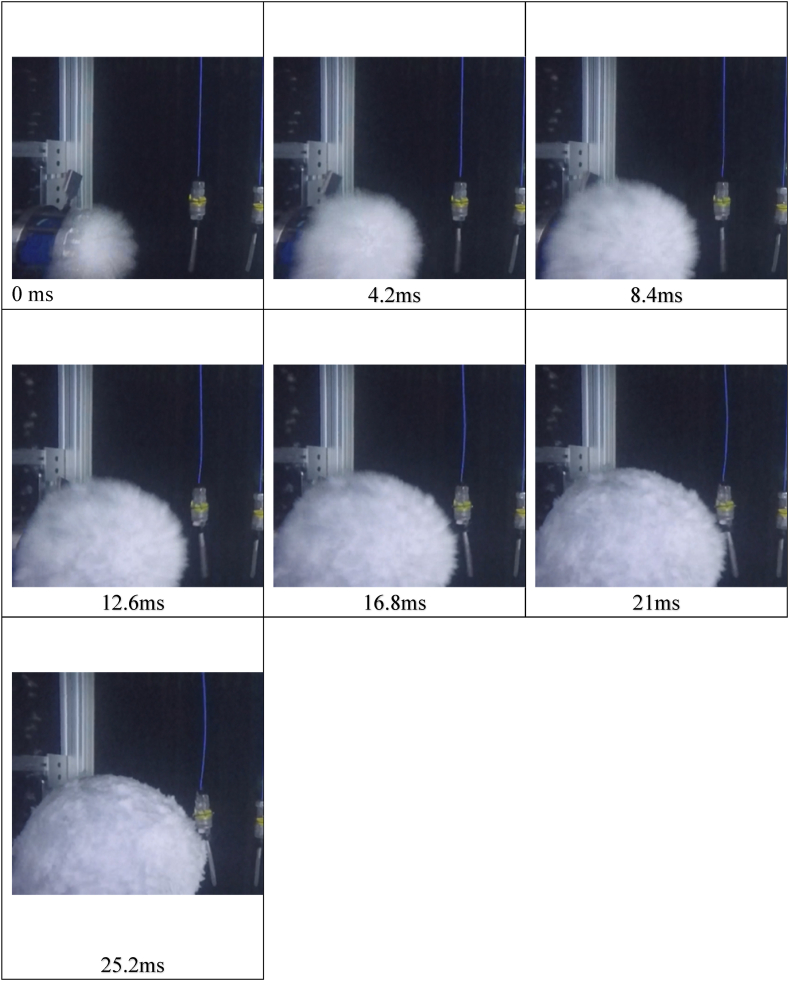
Video frame of bubble expansion.

Shock waves propagated just after the rupture of the disks, and bubbles followed because of the air escaping through the rupture disks. The bubbles expanded and contracted. Fig. 12 shows photos of the bubble expansion taken from a video at specified sequences of time. The pulsation of bubbles produced subsequent shock waves, but those waves were much smaller than the initial shock waves so the maximum pressures in Fig. 10 were identified to have resulted from the initial shock waves.

### 4.2 Carbon fiber composite

The CFC cylinders were fabricated with two layers of dry carbon fiber and epoxy resin mixture. Since both air-back and water-back conditions were studied, Table 5 provides the calculated internal fluid volume for single-wall and double-wall cylinders.

**Table 5:** Internal water volume for CFC cylinders.

Sample Configuration	Water Level [%]	Water Volume [mL]	Water Volume [fl. oz]
Single	0	0	0
Single	50	1795.42	60.71
Single	100	3590.84	121.42
Double	50	1163.35	39.33
Double	100	2326.70	78.67

**Fig. 13:**
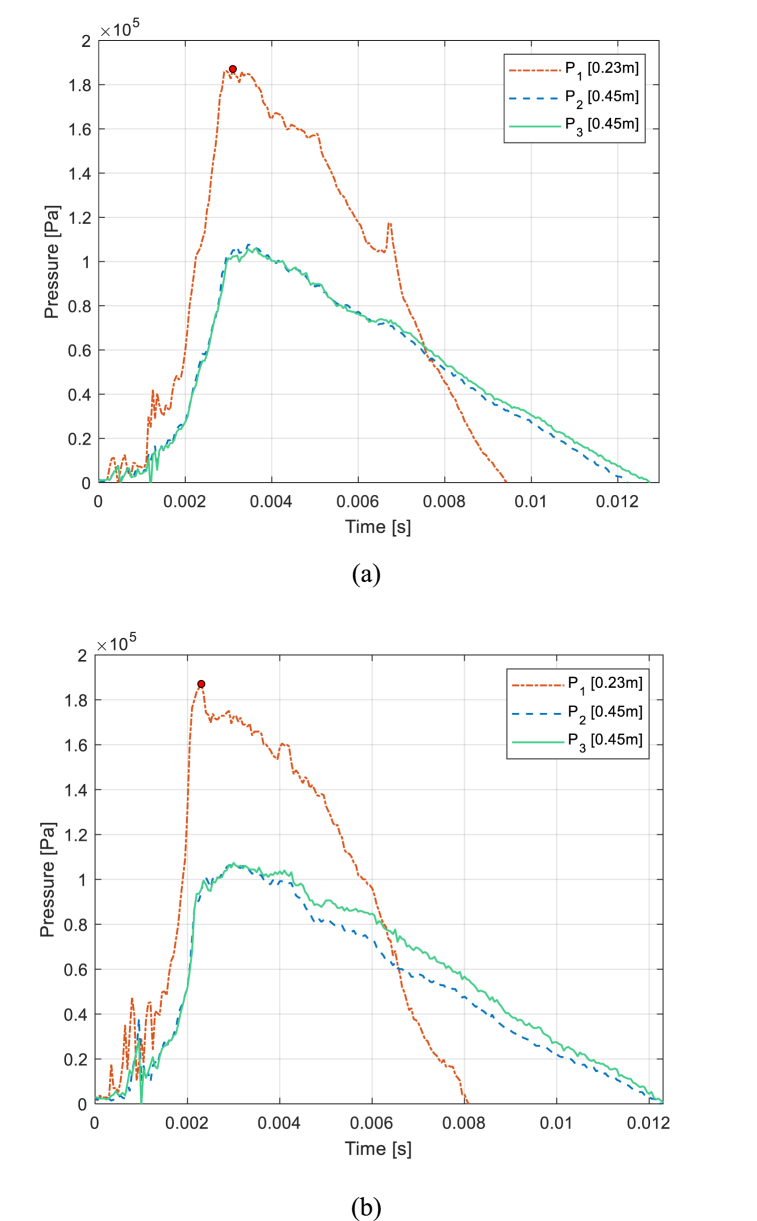
Pressure Profiles for (a) CFC(1) with 0% Water Level and (b) CFC(2) with 100% Water Levels at 0.45 m Stand-off.

CFC cylinders have a starting stand-off distance of 0.45 m (1.47 ft) where the structure does not present any visual failure after loading. In subsequent plots of pressure-time history, the stand-off distances of the pressure sensors are annotated in the legend of the pressure profile plots unless mentioned otherwise. These pressure time histories prove strongly that there is uniformity of the shock wave propagation with P2 and P3 readings almost recording the same data points even if placed at two separate ends of the cylinder, as shown in Fig. 13 (a,b) which shows the pressure time history plots for samples CFC(1) and CFC(2) at 0.45 m (1.47 ft) with 0% water level and 100% water level, respectively. The maximum pressures of both cases coincided perfectly in these test runs.

The CFC(1) sample with 0% water level shows a noticeable reflection wave peak for P_1_ that occurs at approximately 6.7 msec while the CFC(2) sample with 100% water level shows a much lower reflection wave just after 4 msec. This is because the impedance of air is much lower than that of water which is more comparable to that of CFC. Because CFC(1) without internal water was much lighter than CFC(2) with full internal water, and both were freely suspended inside the anechoic water tank; the initial shock wave pushed CFC(1) further from the pressure gage P_1_ than CFC(2). This yielded a delay in the reflection wave peak for the CFC(1) case than the CFC(2) case.

A double-wall cylinder CFC(3–4) at the stand-off distance of 0.3 m (0.98 ft) displays a similar pressure profile as the previous two cases. The CFC(3–4) cylinder with 50% water in the annulus showed the reflection wave much later than the same CFC(3–4) with 100% water in the annulus because of the difference in their weights.

**Fig. 14:**
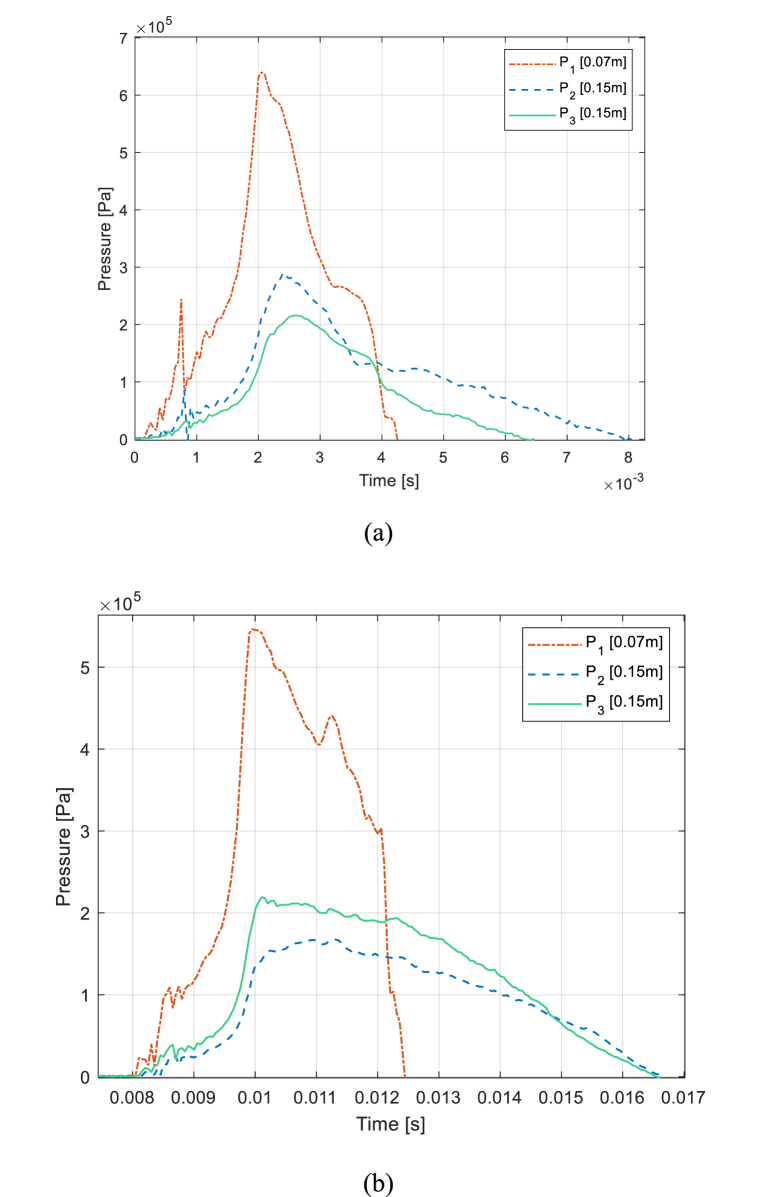
Pressure profiles for CFC(3–4) cylinder sample with (a) 50% and (b) 100% water levels at 0.15 m stand-off.

Fig. 14 (a,b) plots the pressure profiles at a closer stand-off distance of 0.15 m (0.49 ft) for CFC(3–4) between the partially and fully filled annuli. At the distance of 0.15 m (0.49 ft), the pressure reading displays a less polished graph mainly because following the initial burst of the rupture disk are pockets of air that form air bubbles. Where the air bubbles encapsulate the pressure sensors, there are cavities around the sensor. There was a wide variance in the peak pressures and the duration of time for pressure decay from peak to zero.

**Fig. 15:**
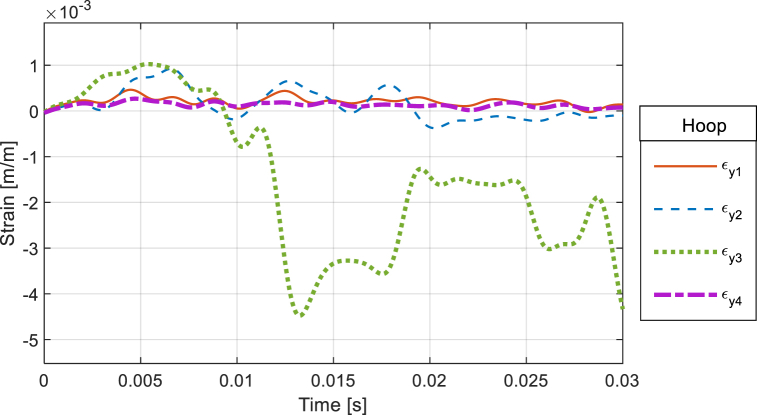
Hoop strain of CFC(1) without internal water at Stand-off Distance of 0.45 m.

The responses of the CFC cylinders were read from the strain gage response. Some strain gages failed during tests, and those readings were not included in the following plots unless mentioned otherwise. Fig. 15 shows the hoop strains of CFC(1) without internal water at the stand-off distance of 0.45 m. The maximum hoop strain was approximately 4.5 *μ* in compression at the posterior side of the cylinder. The reason that the hoop strain was greater at the posterior side than at the front side was due to the motion of the freely suspended cylinder resulting from the shock pressure. As shown in the strain plot, there was no failure of the CFC(1) cylinder without internal water.

**Fig. 16:**
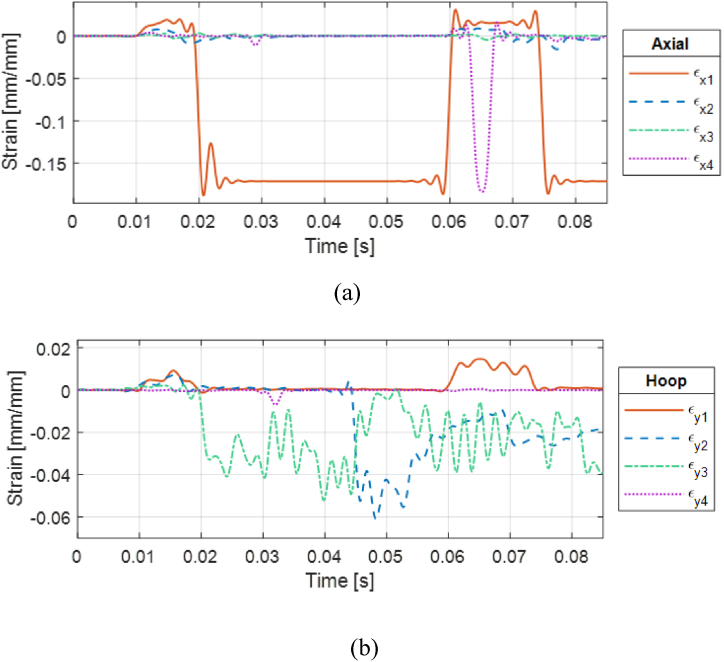
Strain plots for CFC(1) cylinder with 0% water level at 0.21 m stand-off distance: (a) Axial and (b) hoop strains.

**Fig. 17:**
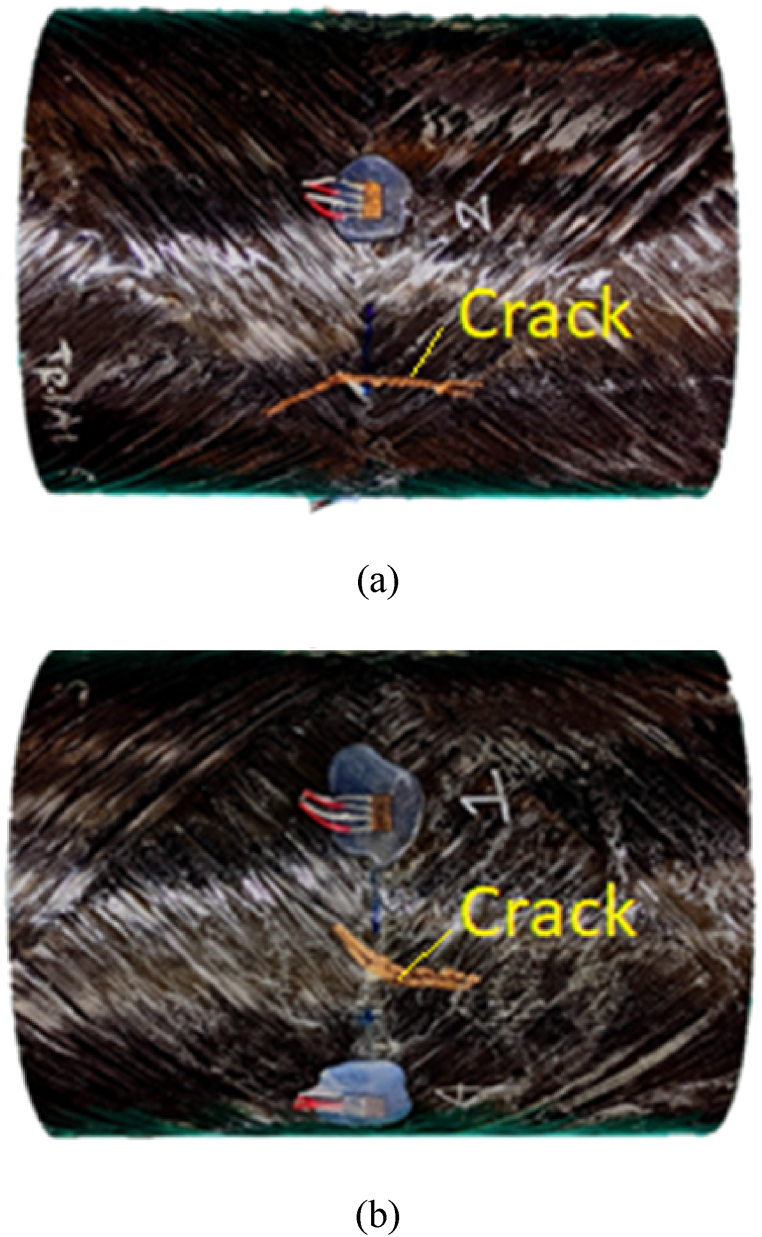
CFC(1) single-wall cylinder with cracks at two locations: (a) Crack between front and bottom, and (b) crack between front and top.

A subsequent test run was conducted at a closer stand-off distance. The CFC(1) sample without internal water fractured at the stand-off distance of 0.21 m (0.68 ft). Fig. 16 (a, b) presents the strain responses of the cracked CFC(1) sample without internal water at 0.21 m (0.68 ft) stand-off distance. The axial strain at the front and bottom sides shows clear failure of the cylinder. This was demonstrated in Fig. 17 (a, b) which shows the cracks at two different locations: one between the front and bottom of the strain gages and the other between the front and top of the strain gages.

The next set of test runs was conducted with CFC(2) composite samples with different water levels: 0%, 50%, and 100%. The 50% water level means the bottom half of the inside of CFC(2) was filled with water while the top half was filled with air. Both axial and hoop strains were compared at the four different locations as sketched in Fig. 3. With 50% water level, the front and posterior strain gages at the level of the internal air/water interface. On the other hand, the bottom strain gage was water-backed while the top strain gage was air-backed.

**Fig. 18:**
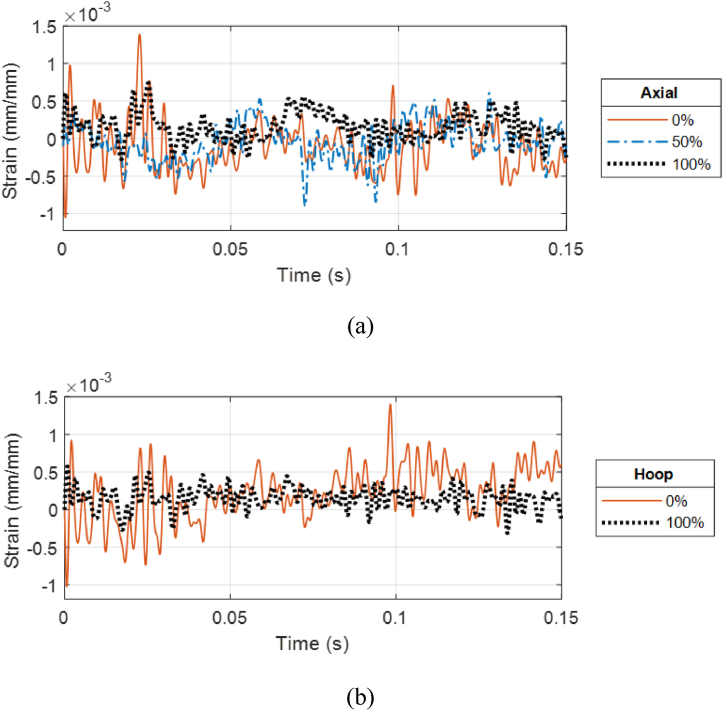
Front strain comparison of CFC(2) single-wall sample for three water levels at 0.45 m stand-off: (a) Axial and (b) hoop strains.

**Fig. 19:**
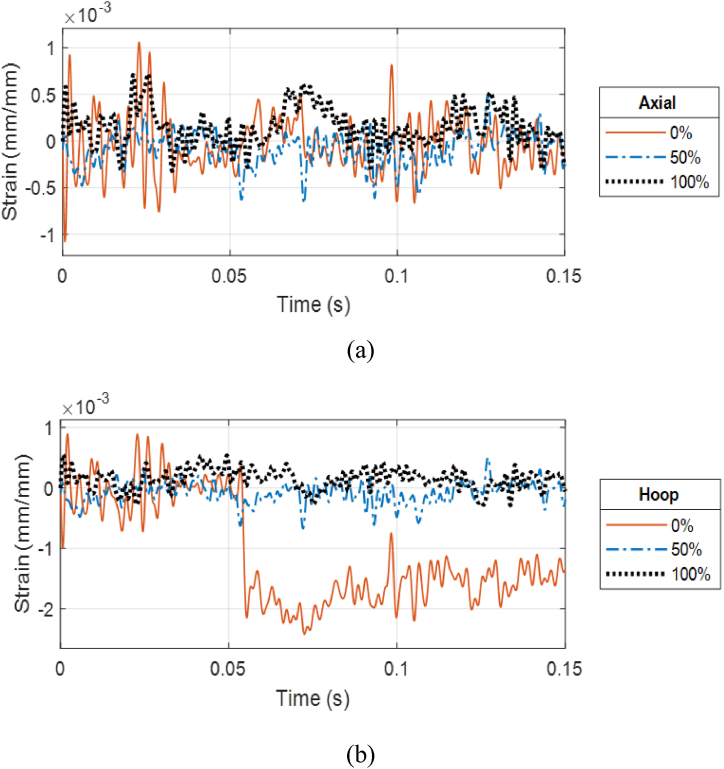
Top strain comparison of CFC(2) single-wall cylinder for three water levels at 0.45 m stand-off: (a) Axial and (b) hoop strains.

Overall, the addition of internal water reduced the strains of CFC(2). Fig. 18 (a, b) and 19 (a, b) show the comparison of both axial and hoop strains at the front and top locations at the stand-off distance of 0.45 m (1.47 ft). In general, 50% and 100% water levels did not produce any significant difference in the strain responses while 0% water was much different from the other two. The case of no internal water of CFC(2) shows a much higher magnitude of hoop strain as compared to the cases of partial and full internal water at the top location as seen in Fig. 19. Even though the 50% water level does not have water just underneath the top strain gages initially, the strain response was similar to that of the 100% water level. This is because the strain response is also influenced by the global deformation of the cylindrical structure. In other words, even though there was no water at the top internal portion of the cylinder with the 50% water level, the bottom portion of water influenced the global deformation of the cylinder and affected the strains at the top of the cylinder. Additionally, sloshing of the internal water in the freely suspended cylinder would result in the top side of the cylinders in continuous contact with internal water.

CFC double-wall cylinders are tested with 15.2 cm (6 in) and 8.9 cm (3.5 in) diameter samples. The water levels specify the amount of water filled within the annulus of the structure, and the inner cylinder is strictly filled with air. CFC(3–4) is analyzed at 100% and 50% water levels only, and 0% was not considered due to the similarity of the single-wall cylinder with the internal air since the interaction of the inner and outer cylinders was considered minimal without water in-between. All strain gage rosettes are attached to the outer surface of the CFC cylinders. Strain gage S1 is located at the front side and S2 is on the posterior side of the outer cylinder, while S3 and S4 are attached in the same manner but for the inner cylinder. The strain responses are examined at stand-off distances of 0.45 m (1.47 ft), 0.30 m (0.98 ft), and 0.15 m (0.49 ft), respectively.

**Fig. 20:**
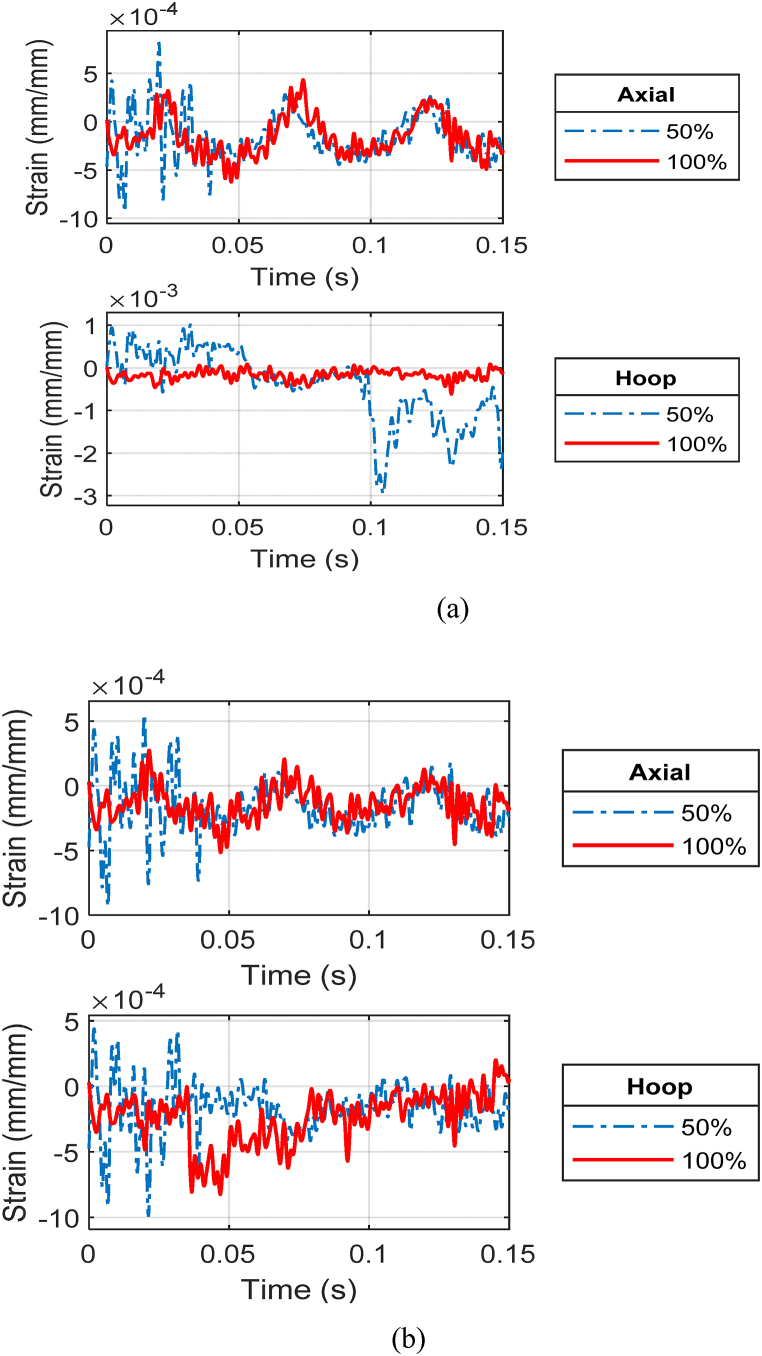
Strain plots of CFC(3–4) at 0.45 m: (a) front gages of outer cylinder and (b) front gages of inner cylinder.

Fig. 20 (a, b) compares the front gage responses of the outer and inner cylinders, respectively, of CFC(3–4) between a partially filled and fully filled annulus at the stand-off distance of 0.45 m (1.47 ft). The partially filled cylinder displayed higher strain responses meaning larger deformation compared to a fully loaded annulus. In addition, the front side strains for both water levels show an in-phase response for the axial directions meaning that the cylinder moves at the same frequency regardless of the internal fluid. The axial strains at later times were very close between the 50% and 100% water cases. The water level influenced hoop strains more than axial strains.

**Fig. 21:**
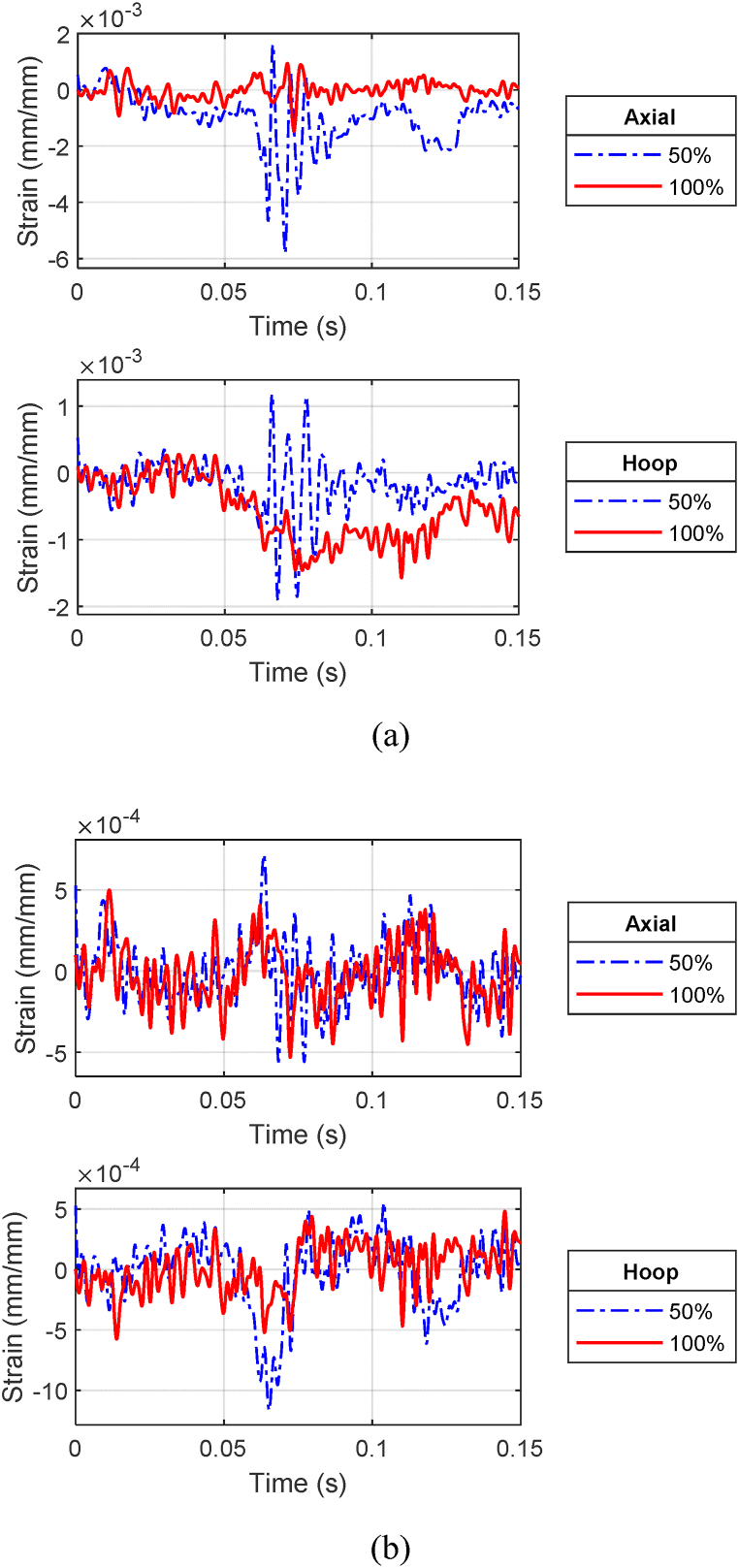
Strain plots of CFC(3–4) at 0.3 m: (a) front gages of outer cylinder and (b) front gages of inner cylinder.

**Fig. 22:**
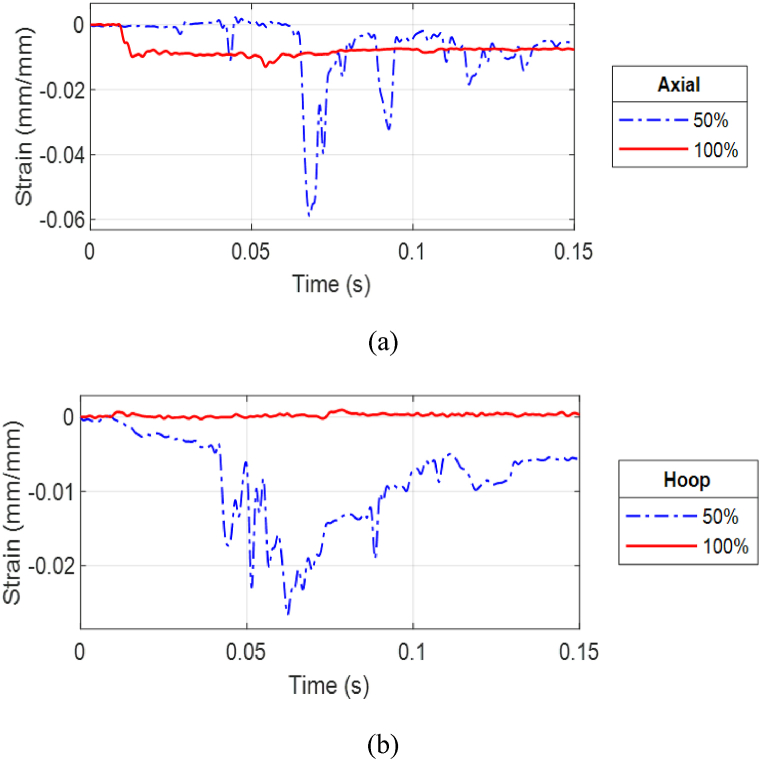
Strain plots of CFC(3–4) at 0.3 m at the posterior site of the inner cylinder: (a) axial and (b) hoop strains.

As the stand-off distance of CFC(3–4) was decreased to 0.3 m (0.98 ft), the strain responses between the partial and full internal water cases became more different for the front side of the outer cylinder and much less for the front side of the inner cylinder, as shown in Fig. 21 (a, b). The axial strain at the front of the outer cylinder was much greater for the 50% water case than for the 100% water case. That is, the effect of the water level became more critical as the stand-off distance became closer. The same statement is also true for the strain response at the posterior side of the outer cylinder. However, the most significant impact of the water level was noticed at the posterior side of the inner cylinder. The difference in the strains at that location was significant as shown in Fig. 22(a, b). Even though the maximum axial strain was up to 0.06 for the 50% water case, there was no visible damage in the inner and outer cylinders of CFC(3–4).

The 50% water level resulted in a structural failure at 0.15 m (0.49 ft) for the CFC(3–4) cylinder. Strain responses suggest failure, and multiple localized cracks were observed on the outer CFC(3–4) cylinder. Fractured CFC cylinders were affected most in the front and/or top sides of the structure, describing a local failure, and no other failure was detected in a post-fracture visual inspection. Moreover, the 100% water level did not show failure strain responses.

### 4.3 Polylactic acid composite

All PLA cylinders are created using the same print settings as previously mentioned, and the one difference is the filament colors used which are PLA Silver Metallic, PLA Green, and PLA Blue. The different colors of PLA have the same material properties. The cylindrical structures consist of a 15.2 cm (6 in) diameter for the single-wall cylinders, and the double-wall cylinder has a 15.2 cm (6 in) outer diameter with a 12.7 cm (5 in) inner diameter. The internal fluid volumes are calculated for both single and double-wall cylinders and given in Table 6.

**Table 6:** Internal water volumes for PLA cylinders.

Sample Configuration	Water Level [%]	Water Volume [mL]	Water Volume [fl. oz]
Single	0	0	0
Single	50	1795.42	60.71
Single	100	3590.84	121.42
Double	50	556.58	18.82
Double	100	1113.16	37.64

**Fig. 23:**
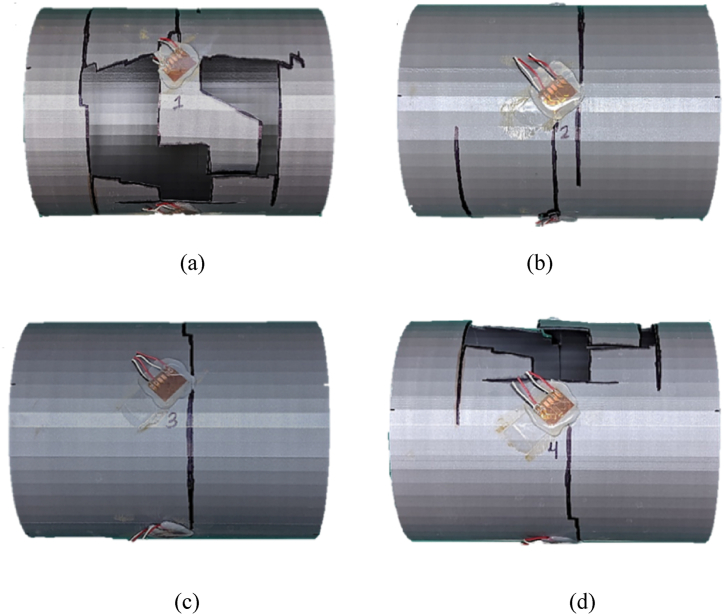
PLA(1) Single-wall cylinder sample with ruptured areas outlined (a) front side (b) top side (c) posterior side (d) bottom side.

A single-wall PLA cylinder with 0% water level showed a structural failure at 0.75 m (2.46 ft) stand-off distance, which suggested that the maximum shock load tolerance for the single-wall PLA was beyond 0.75 m (2.46 ft) stand-off distance. The PLA cylinder without internal water had a sudden rupture because the material is brittle. The sudden break made the cylinder into multiple pieces as shown in Fig. 23(a–d).

As a result, 100% water levels for single and double-wall configurations were tested from there on out while using the 0% water level test strain data as a valuable comparison. With the limited number of rupture disks available in the lab during this study, only one test run for each stand-off distance at 0.75 m (2.46 ft), 0.60 m (1.97 ft), and 0.45 m (1.47 ft) was performed for each cylindrical configuration.

The PLA cylinders with 100% water levels had a starting stand-off distance at 0.75 m (2.46 ft) where the structures did not present any failure after shock loading confirming internal water increased the shock load tolerance as compared to 0% water level cylinders. Next, PLA(2) was tested with 100% water at 0.60 m (1.97 ft), respectively. The cylinder with full internal water did not fail at 0.60 m (1.97 ft).

**Fig. 24:**
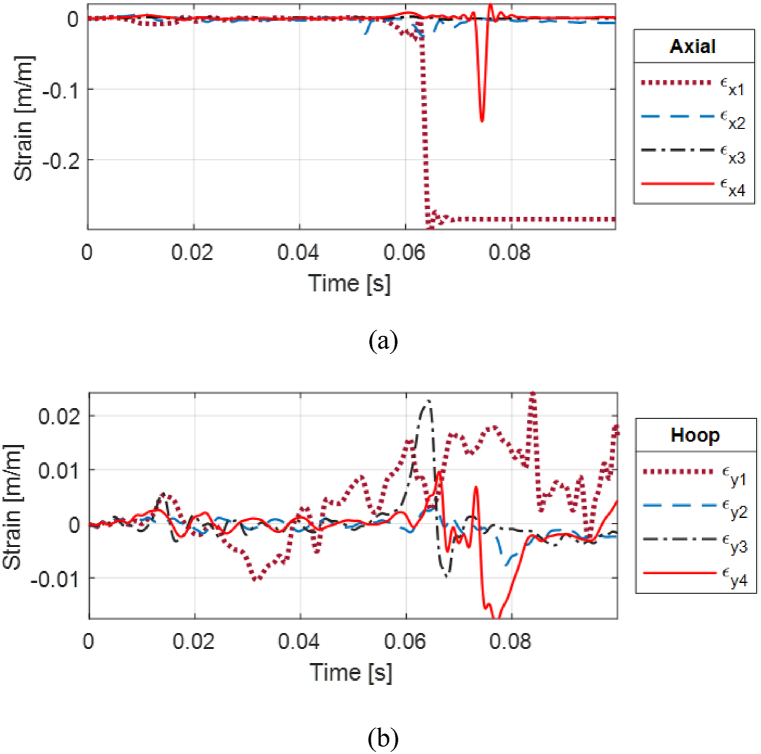
Fractured strain response of PLA(1) single-wall cylinder with 0% water level at 0.75 m stand-off: (a) Axial and (b) hoop strains.

Strain responses of two cases are presented with various stand-off distances for PLA cylinders. Fig. 24(a, b) presents the strain for the PLA(1) cylinder which shows a clear fractured response by the magnitude and characteristics of the strain. Much of the shock load was absorbed by the front strain gauge rosette which shows the highest magnitude compared to the top, posterior, and bottom locations, as seen in Fig. 23.

**Fig. 25:**
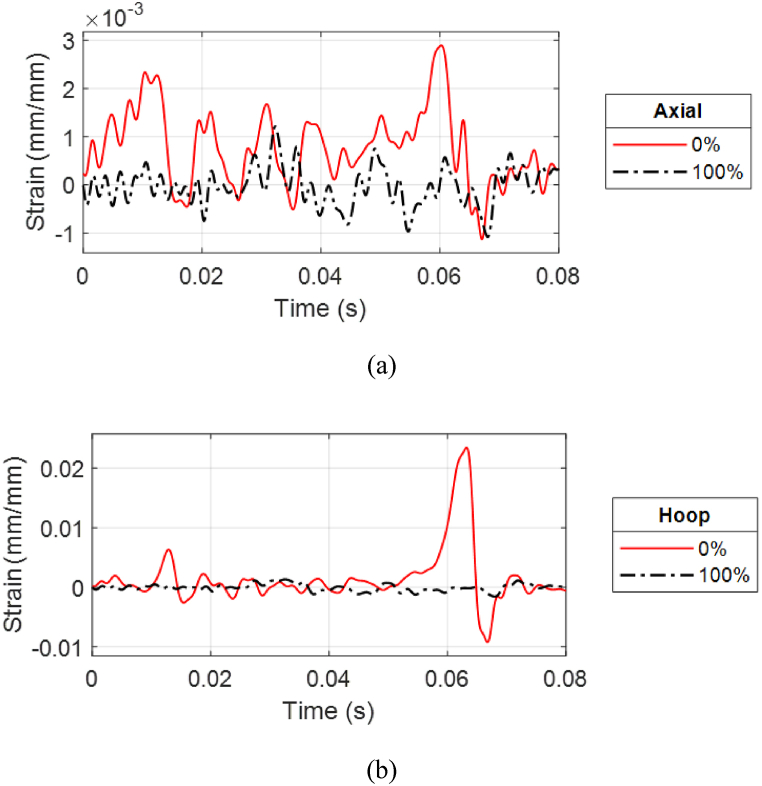
Posterior strain comparison of PLA cylinders for two water levels at 0.75 m stand-off: (a) Axial and (b) hoop strains.

The strain responses were compared between PLA(1) with 0% water and PLA(2) with 100% water. They were significantly different at all strain gage locations. Fig. 25(a, b) compares the axial and hoop strains at the posterior side of the cylinders. Internal water significantly reduced the strain response of the PLA cylinder to prevent failure because water has a more comparable speed of sound than air as compared to PLA. In addition, the internal water provided added support to the cylinder.

**Fig. 26:**
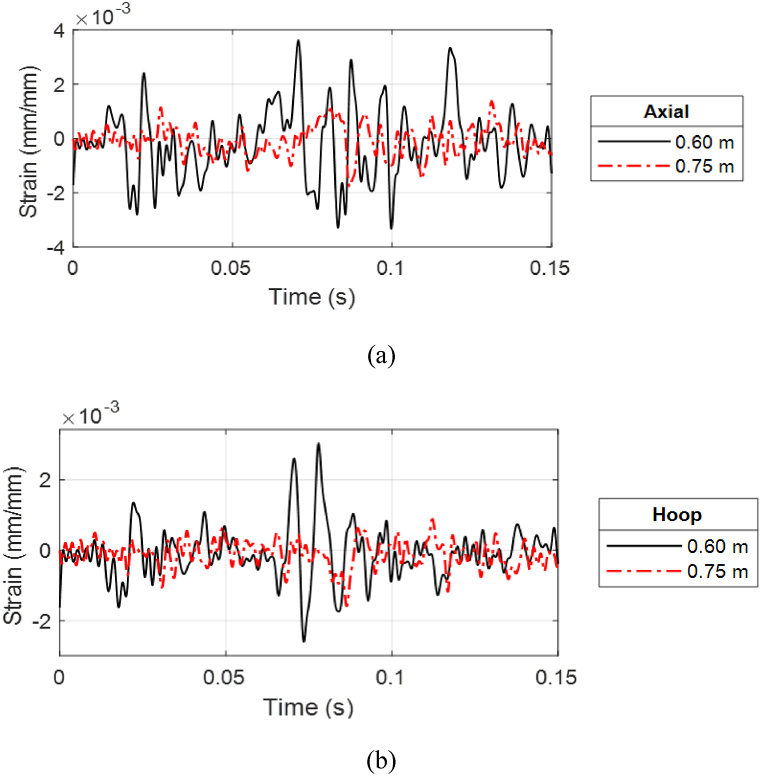
Top strain comparison of PLA(2) for two stand-off distances with 100% water level: (a) Axial and (b) hoop strains.

As expected, the strain response at 0.60 m (1.97 ft) is much larger than that of 0.75 m (2.46 ft) because of the higher pressure, as shown in Fig. 26(a, b). The hoop strain at 0.60 m (1.97 ft) captured the movement of the shock wave as it showed an increase in strain response followed by a decrease as the wave passed through. The different stand-off distances produced not only different magnitudes but also different characteristics such as frequencies of the strain responses for the same cylinder because the pressure-time history varies depending on the stand-off distance.

**Fig. 27:**
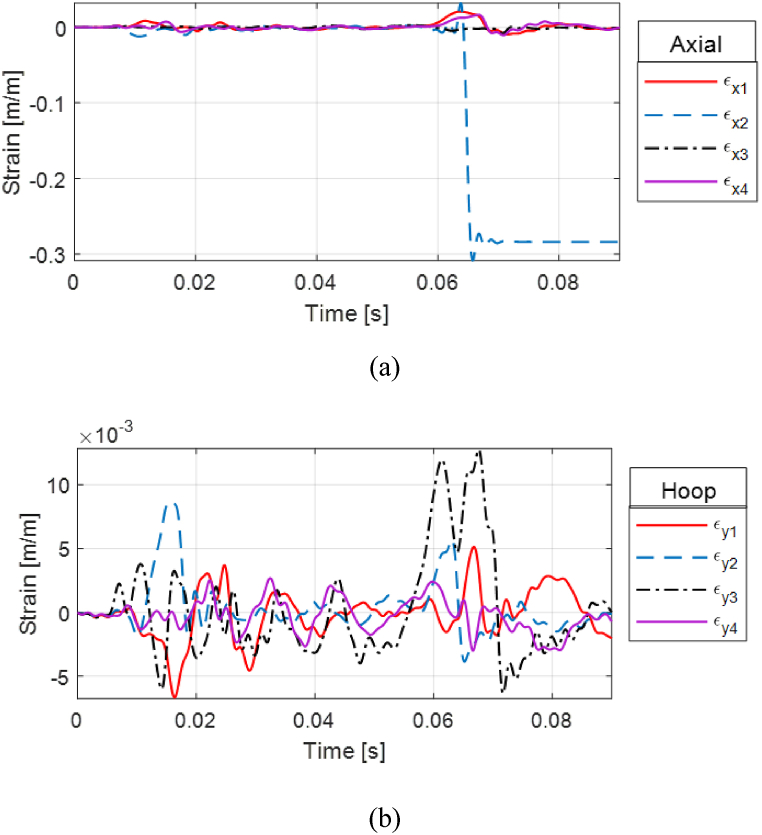
Strain response of failed PLA(2) single-wall cylinder with 100% water level at 0.45 m stand-off: (a) Axial and (b) hoop strains.

To the next testing sequence, PLA(2) was then moved to the closer stand-off distance where a PLA cylinder is expected to rupture at 0.45 m (1.47 ft). Fig. 27(a, b) presents the strain plot of PLA(2) at 0.45 m (1.47 ft) with 100% water level, where the fractured strain response can be seen as a steep rise of the axial strain at the top side.

**Fig. 28:**
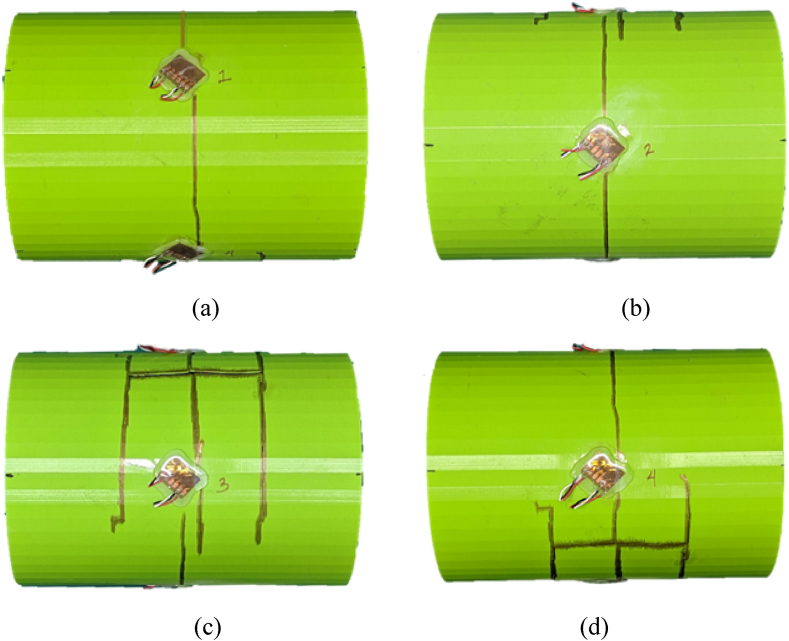
PLA(2) cylinder with ruptured areas outlined at (a) front side, (b) topside, (c) posterior side, (d) bottom side.

Fig. 28(a–d) displays the ruptured PLA(2) sample from the front to the bottom side with the outlined fractured areas. The shock loading had a larger effect on the posterior side than on the front side. Shock wave hitting the front side of the cylinder is transmitted into the internal water because of the similar impedance of the water and the cylinder material. The transmitted shock wave reaches the posterior side of the cylinder which is also subjected to shock pressure via the surrounding outer water. Such multiple shock waves seem to result in a greater effect on the posterior side.

PLA(3–4) samples with 100% water were tested at 0.75 m (2.46 ft), 0.60 m (1.97 ft), and 0.45 m (1.47 ft) as a double-wall cylindrical structure. Unfortunately, the strain data at the 0.75 m (2.46 ft) stand-off distance was not recorded correctly and is not a viable source for comparison. However, other test runs were successful and strain data were properly collected.

**Fig. 29:**
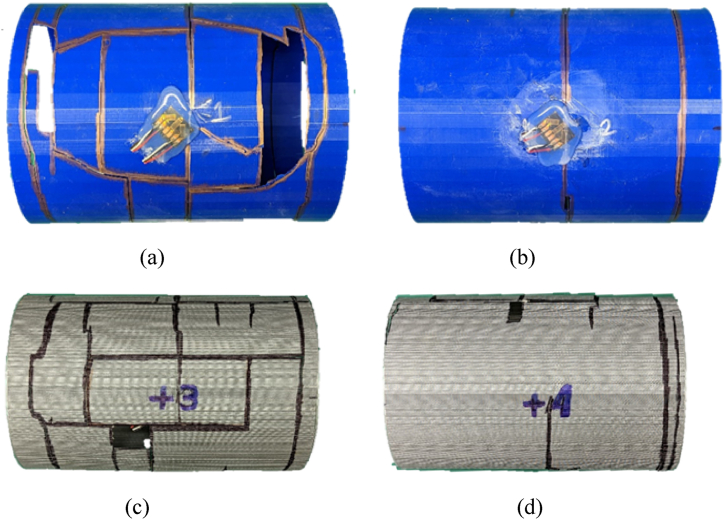
Ruptured PLA(3–4) double-wall sample with fractured areas outlined: (a) front side of outer cylinder, (b) posterior side of outer cylinder, (c) front side of inner cylinder, and (d) posterior side of inner cylinder.

PLA(3–4) with 100% water failed at 0.45 m (1.47 ft) stand-off distance. Fig. 29(a–d) displays the outer (left) and inner (right) cylinders for the PLA(3–4) sample with the fractured areas outlined. Both inner and outer cylinders failed much greater at the front sides than at the posterior sides. This was different from the single-wall cylinder with 100% water, which showed failure at the posterior side. In other words, the inner cylinder with water in the annulus made the failure of the outer cylinder at the front side like a cylinder without water.

## 5 Summary and conclusions

Both polymeric CFC and PLA cylinders were investigated for their responses when subjected to underwater shock loading by conducting experiments inside an anechoic water tank using the CASPUR to generate shock pressure. The CFC cylinders had winding angles of ±45°. The cylinders had two different configurations: single-wall and double-wall. The latter has two cylinders of different diameters which are arranged concentrically so that there is a uniform spacing between the two cylinders. Both single-wall and double-wall cylinders had different water amounts inside of them. The double-wall cylinders had water only in the annulus but not inside the inner cylinder. The amount of water was represented by 0%, 50%, or 100% of the volume where water was filled. The case of 0% means there is no water such that it is an air-back condition. All the tested cylinders were suspended freely inside the water. To overcome the buoyancy forces so that the tested cylinders remained at their specified location, a weight was attached to the cylinders by strings.

The stand-off distance varied from a farther distance to a closer distance incrementally until the cylinders failed. Both shock pressures and strain responses were measured in every experiment. Three pressure gages were used for each test. One pressure gage was located at half the stand-off distance of the tested cylinders, while two pressure gages were placed on both sides of the cylinders, respectively, at the same stand-off distance. The former pressure gage checked the progress of the shock wave, and the two other pressure gages were used to represent the shock pressure arrived at the tested cylinders. Strains were measured in four different locations for each test. Both axial and hoop strains were measured at each site. The locations for single-walled cylinders were the front, top, posterior, and bottom of the cylinders, which were positioned at the centerline along the direction of their length. The double-wall cylinders had strain gages only at the front and posterior sites of both the inner and outer cylinders.

The pressure profiles created by the CASPUR system were quite consistent so that a fair comparison could be made from test to test. The maximum pressure was a function of stand-off distance, and the plot of the maximum pressure vs. distance was well curve-fitted using a linear inverse function of the stand-off distance as expressed in Eq. (1). The pressure propagated symmetrically such that pressure gages at both sides of tested cylinders recorded comparable pressure regardless of the number of broken pedals in rupture disks.

The inclusion of water inside both single-wall and double-wall cylinders increased the resistance against failure due to underwater shock loading. Among all the cases, 100% water made the structure strongest against underwater shock loading. The strain response of 50% water was much closer to that of 100% water than that of 0% water. Two major factors contributed to the last statement. One was that partially filled internal water in the freely suspended cylinder has a sloshing effect to wet the whole cylinder surface similar to the full water. The other was that partially filled water influences the global deformation of the cylinder.

An impact study on concentric cylinders with water in the annulus suggested that an internal water level of 25% or less had a negligible effect of the deformation of the cylinders [28]. However, because the study in Ref. [28] used an impact loading on the cylinders in air, a further study will be necessary to confirm that in the present cases with different types of loading and surrounding fluid media because those would make a major difference in the dynamic responses and failure.

Major failure sites depended on the number of cylinders (i.e., single-wall or double-wall) as well as internal water. For single-wall cylinders without internal water, major failures like cracks or ruptures occurred on their front sides. When single-wall cylinders had internal water, failure was more dominant on the posterior sides.

The internal water affects the shock wave propagation in the cylinder. Because the impedance of the water is closer to that of the cylinder than air, shock wave more readily transmits into the water with less reflection. This made the front side of the cylinder less vulnerable to failure. Instead, the posterior side of the cylinder is subjected to multiple shock waves through the outside water, internal water, as well as the cylinder itself. This made the transition of the major failure location from the front to the posterior site with internal water.

The double-wall cylinders had a major failure with water at the front sides of the cylinders. This was similar to the single-wall cylinder without internal water. The wave interaction of the double-wall cylinder is much more complex than the single-wall cylinder. A numerical study will be conducted to properly understand the failure of the double-wall cylinders. Double-wall CFC cylinders only had cracks in the outer cylinder while double-wall FLA cylinders showed failure of both inner and outer cylinders because CFC cylinders are much stronger than the FLA cylinders.

## Data availability statement

The raw/processed data required to reproduce these findings cannot be shared at this time as the data also forms part of an ongoing study. Most of the data are included in the present paper.

## CRediT authorship contribution statement

**Vernajo P. Macapagal:** Writing – original draft, Validation, Investigation, Data curation. **Young W. Kwon:** Writing – review & editing, Writing – original draft, Validation, Supervision, Funding acquisition, Conceptualization. **Jarema M. Didoszak:** Writing – review & editing, Validation, Supervision, Resources.

## Declaration of competing interest

The authors declare the following financial interests/personal relationships which may be considered as potential competing interests: Young Kwon reports financial support was provided by 10.13039/100000006Office of Naval Research. If there are other authors, they declare that they have no known competing financial interests or personal relationships that could have appeared to influence the work reported in this paper.

## Nomenclature

3-D: three-dimensional
CFC: carbon fiber composite
CASPUR: compressed air shock pipe underwater release
DAQ: data acquisition
FSI: fluid-structure interaction
NPS: Naval Postgraduate School
NI: National Instrument™
PLA: polylactic acid
RTV: room temperature vulcanizing
UNDEX: underwater explosion
